# Reciprocal Relationships Between Moral Competence and Externalizing Behavior in Junior Secondary Students: A Longitudinal Study in Hong Kong

**DOI:** 10.3389/fpsyg.2019.00528

**Published:** 2019-03-06

**Authors:** Daniel T. L. Shek, Xiaoqin Zhu

**Affiliations:** Department of Applied Social Sciences, The Hong Kong Polytechnic University, Hung Hom, Hong Kong

**Keywords:** delinquent behavior, problem behavioral intention, virtue, reciprocity, Chinese adolescents

## Abstract

Defining moral competence using a virtue approach, this longitudinal study examined the prospective relationships between moral competence and externalizing behavior indexed by delinquency and intention to engage in problem behavior in a large and representative sample of Hong Kong Chinese adolescents. Starting from the 2009–2010 academic year, Grade 7 students in 28 randomly selected secondary schools in Hong Kong were invited to join a longitudinal study, which surveyed participating students annually during the high school years. The current study used data collected in the first 3 years (Wave 1 to Wave 3) across junior secondary school stage (Grades 7–9) with a sample of 3,328 students (Age = 12.59 ± 0.74 years and 52.1% boys at Wave 1). Cross-lagged panel path analyses were conducted to compare four models involving different hypothesized patterns of relationships between moral competence and externalizing behavior. Results revealed that the reciprocal effects model best fit the data, supporting reciprocal causal relationships between moral competence and externalizing behavior measures. Specifically, a higher level of moral competence significantly predicted a lower level of delinquency and problem behavioral intention over time. At the same time, a higher level of externalizing behavior also significantly predicted a lower level of moral competence 1 year later. As the magnitudes of the significant findings were not high, replications in different Chinese communities are needed. Nevertheless, the present findings provide important theoretical insights on how moral competence and externalizing behavior in adolescents are associated with each other. Practically speaking, the findings suggest that it is promising to reduce adolescent externalizing behavior by promoting their virtues through moral education programs, and guiding adolescents to behave in a good manner would help promote the development of their virtues.

## Introduction

While there are theoretical propositions and empirical evidence on the impacts of moral competence (indexed by virtues) on adolescent externalizing problems such as delinquent behavior (Catalano et al., 2002; Seligman, 2002), there are also indicators suggesting the reciprocal relationship between moral competence and delinquency (Matsueda, 1989; Raaijmakers et al., 2005). Unfortunately, there are research gaps in regards to existing literature. First, compared with ample investigations on the features and patterns of adolescent moral virtues as well as the linkage between virtues and adolescent well-being, empirical research addressing relationships between moral competence and delinquency is inadequate. Second, few studies have used a large sample size and longitudinal data to answer the related questions. Third, few studies have examined the related issues among non-Western populations such as Chinese adolescents. Hence, the present paper attempts to address these research gaps using a large-scale longitudinal design in Hong Kong.

### Defining Adolescent Moral Competence as Virtues

The literature in the research area of moral development has come to agree with James Rest (1994) view that morality is a multidimensional concept which includes moral sensibility, moral judgment, moral motivation, moral identity, and moral character. There are two main approaches covering these concepts: one is the social-cognitive framework related to Kohlberg’s original theory on moral reasoning (Kohlberg, 1964) and the other is the virtues approach which has received very much attention recently from researchers based on the positive psychology approach (Catalano et al., 2002; Park and Peterson, 2006). The former one conceives moral competence as the ability to make moral judgments and behave accordingly (Kohlberg, 1964). According to this framework, one specific cognitive process of moral judgment is moral reasoning which represents the pattern along which a concrete argument or decision is produced (Kohlberg, 1984). Basically, as an individual grows from a child to an adult, his or her moral reasoning ability develops along a six-stage process from a lower stage characterized by egocentric thinking to higher ones involving more social oriented thinking (Gibbs, 2014). In literature, moral reasoning ability is usually assessed by objective dilemma-based moral judgment interviews or dilemma-free production tests (Gibbs et al., 2007). The research on the development of moral judgment competence and its association with adolescent development has been well established in several cultures including China (Stams et al., 2006; Gibbs et al., 2007; Zhang and Yang, 2013; Cheng, 2014; Lind, 2017; von Grundherr et al., 2017). For example, It is well known that delayed development of moral reasoning would put adolescents at a higher risk of developing delinquent behavior (Kohlberg, 1984; Gibbs et al., 2007).

Using virtues approach, scholars in the field of positive psychology define adolescent moral competence as a trait-like construct. For example, Park and Peterson (2006, p. 891) stressed that “moral competence among the adolescents can be approached in terms of good character,” which represents a set of positive traits widely and historically valued by the society. Accordingly, they proposed Values in Action (VIA) Classification framework that consists of six core virtues which further cover 24 character strengths, such as “creativity,” “loving of learning,” “social intelligence,” “self-regulation,” and “appreciation of beauty and excellence.” Accordingly, a comprehensive self-report VIA Inventory of Strengths for Youth (VIA-IS) including 198 items have been developed to measure 24 strengths of character in adolescents (Park and Peterson, 2006). The universal occurrence of virtues and character strengths and their developmental characteristics among adolescents have been sufficiently supported, especially in Western countries such as United States, United Kingdom, and Canada (Dahlsgaard et al., 2005; Park et al., 2006).

Similarly, scholars in the positive youth development (PYD) field consider adolescent moral competence as “a sense of right and wrong or a sense of moral or social justice” (Catalano et al., 2002, p. 19) and rate it as one of fifteen critical PYD constructs (e.g., cognitive competence, social competence, emotional competence, and spirituality, etc.) that promote desired youth developmental outcomes (Catalano et al., 2002). Different from the VIA framework where moral virtues cover broad positive traits in multiple dimensions, Catalano et al. (2002) 15 PYD constructs framework separates moral competence from other psychosocial competencies (e.g., “cognitive competence,” “spirituality,” and “social competence”). In other research areas, such as management field, scholars have also defined moral competence from the virtue perspective. For instance, Morales-Sánchez and Cabello-Medina (2013, p. 721) stated that “moral virtues in the workplace would be moral competencies.” Martin and Austin (2010) argued that moral competence can be indexed by ten positive traits such as active caring about others and keeping promises.

Despite consensus on the importance of adolescent moral virtues, studies found different virtue structures in different cultures. For instance, a four-factor structure (Brdar and Kashdan, 2010) and a three-factor structure (Duan et al., 2012b) were observed in Croatia and China, respectively. Besides, a recent study identified different profiles between Chinese and Western adolescents on some character strengths (Liu et al., 2016). Specifically, while Western adolescents scored higher on kindness, gratitude, curiosity than on persistence, modesty, and self-regulation (Park et al., 2006), Chinese adolescents scored higher on self-regulation, modesty, and persistence than on humor, graduate, and curiosity (Liu et al., 2016). These findings suggest that it is important to take cultural context into account when studying moral competence.

As Chinese people constitute approximately one-fifth of the world’s population, scholars argued that “if any theory is claimed to be universally applicable, relevant data from Chinese people must be collected” (Shek, 2006, p. 276). Thus, studying the relationship between moral virtues and developmental outcomes among Chinese adolescents is indispensable to shed light on the universality of such a relationship. Noteworthy, Chinese people’s beliefs and social life patterns are profoundly shaped by traditional Confucian philosophy which stresses on society’s moral order, harmonious interpersonal relationships, and individuals’ cultivation of moral virtues (Shek et al., 2013; Ma and Tsui, 2015). Therefore, it is advocated that Chinese adolescent moral competence should be defined with reference to traditional Confucian virtues (Shek et al., 2015). In line with this notion, in their measurement of Chinese adolescent positive development framed under Catalano et al. (2002) 15 PYD constructs framework, Shek et al. (2007) included moral competence based on Confucian virtues.

There are three virtues that deserve particular attention because they have been historically emphasized in Chinese societies and also echo the character strengths under the Western conception. The first virtue has to do with the general Confucian thought of striving to be “a perfect and virtuous man” (“*Junzi* 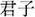
” in Chinese), which implies high moral expectation for oneself (Chan, 2008). Influenced by this notion, education in China strongly emphasizes that one should have high moral standards for oneself as manifested in one’s thoughts, feelings and behaviors (Wang, 2004; Gu, 2006). This virtue is conceptually similar to the character strengths such as “appreciation of beauty and excellence” and “courage” in the VIA framework (Park and Peterson, 2006).

The second virtue is trustworthiness (“*xin* 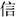
” in Chinese), which is derived from “keeping one’s promise” and further highlighted as one of the five Confucian cardinal virtues (Shek et al., 2013). This virtue is so important that it is mentioned nearly 40 times in The Analects. The “*xin*” in Chinese culture echoes the character strengths of “persistence in one’s words and behaviors” in the VIA model (Park and Peterson, 2006). The “*xin*” or trustworthiness is also a manifestation of a sense of responsibility (Jormsri et al., 2005) and constitutes an effective approach for building and maintaining mutual trust in social interactions (Schniter et al., 2013).

The third virtue is about self-evaluation and self-reflection. Confucianism emphasizes promoting one’s morality through evaluating and reflecting on oneself continuously and frequently (Wang, 2004). This virtue is closely linked to “authenticity” and “self-regulation” in Park and Peterson (2006) framework of character strengths and is subsumed under “prudence” in Morales-Sánchez and Cabello-Medina (2013) moral competency model.

### Moral Competence and Externalizing Behavior: Direction of the Relationship

Within the virtues approaches, it is argued that a good life characterized by happiness, life satisfaction and absence of problem behavior can be constructed by promoting strengths of character among the youth, as good character will direct one’s thoughts and behavior to do good things effectively (Catalano et al., 2002; Park and Peterson, 2006). In line with this theoretical assertion, existing evidence generally demonstrated that good characters are closely associated with youth well-being and favorable outcomes (Peterson et al., 2007; Gillham et al., 2011; Duan et al., 2012a; Quinlan et al., 2012). For example, characters strengths such as love, hope, perseverance were the robust predictors of life satisfaction among Western adolescents (Peterson et al., 2007; Martínez-Martí et al., 2016). Character strengths were also positively associated with other favorable outcomes, such as resilience and coping strategies (Gustems-Carnicer and Calderón, 2016; Martínez-Martí and Ruch, 2017). Likewise, Chinese virtues were positively associated with life satisfaction and flourishing among university students in Mainland China and Hong Kong (Duan et al., 2012a; Duan and Ho, 2018).

There is no doubt that moral virtues are essential for adolescents to thrive. While a number of studies have investigated virtues’ contribution to adolescent well-being indexed by happiness or life satisfaction (e.g., Peterson et al., 2007; Gillham et al., 2011), only a few empirical studies demonstrated that certain characters (e.g., self-regulation) negatively predicted the youth problems (Kim and Brody, 2005; de Kemp et al., 2009). Overall speaking, the relationship between moral virtues and adolescent externalizing behavior has not been adequately studied, particularly in the Chinese context where research on moral competence based on the virtue approach is still at its infancy. In Chinese communities, the topic of moral virtues among adolescents has begun to receive academic research attention recently (Liu et al., 2016). Besides, most existing studies focused on developing and validating measurement tools (e.g., the Chinese version of VIA-IS) or investigating virtues patterns of moral characters (e.g., profile and virtue-structure) among Chinese youths (Duan et al., 2012b, 2013; Liu et al., 2016; Duan and Bu, 2017). In contrast, few studies have investigated the relationship between moral virtues and developmental outcome indicators. Although few recent studies have related Chinese virtues to individuals’ psychological well-being (Duan et al., 2012a, 2013; Duan and Guo, 2015; Zhang and Chen, 2018), they all focused on university students (i.e., late adolescents and young adults). To conclude, similar to the situation in Western contexts, the relationship between Chinese virtues and externalizing behavior among adolescents remained severely under-researched.

In addition, among the few exceptions, findings on the association between moral virtues and adolescent problem behaviors are not conclusive. For example, Kim and Brody (2005) found the longitudinal predicting effect of self-regulation on the externalizing behavior consisting of aggressive and delinquent behavior. A recent study reported a negative association between moral virtues and smartphone addiction among Chinese university students (Lian et al., 2016). The authors argued that virtues serve as positive psychological assets that help adolescents to control their negative behavior. Likewise, the present authors’ prior work also showed that moral competence in terms of virtues was significantly related to the initial level and developmental trajectory of externalizing problems among Chinese adolescents in Hong Kong (Shek and Zhu, 2018). However, in Seider et al. (2013) study, students’ perseverance and integrity did not show significant predictive effects on their delinquent behavior in the regression analyses, although the two virtues were reversely correlated with delinquency.

So far, most discussions have concentrated on the possible causal effect of moral virtues on the adolescent externalizing behavior while almost no research has ever considered an alternative perspective that one’s externalizing behavior itself may exert an impact on the (lack of) development of moral competence. Yet, there are some indications of such a plausibility in the previous research. For example, Matsueda (1989) study demonstrated that the adolescent delinquent behavior exerted an even more substantial effect on future moral beliefs (e.g., one believes that not cheating is a good thing to do) than did the moral beliefs affect future delinquency. Moral beliefs and moral virtues are different concepts, yet we might still expect comparable relationships between moral virtues and delinquency, given that moral beliefs and moral virtues are both related to morality development and somehow, they are related to each other. For example, it makes sense to speculate that a virtuous individual (e.g., having high moral expectations for oneself and being trustworthy) would have a stronger belief in the moral order (e.g., one should not cheat or lie). Likewise, an individual holding strong moral beliefs is more likely to develop virtues which fit one’s beliefs. More recently, moral reasoning, another concept of morality which represents a conceptualization of moral competence under the social-cognitive framework, was found to have a reciprocal causal relationship with delinquency among adolescents and young adults (Raaijmakers et al., 2005). This finding serves as another indirect support for the effect of delinquency on moral virtues, another conceptualization of moral competence using the virtues approach.

There are several possible underlying mechanisms for the influence of externalizing behavior on virtues. First, some scholars argued that one’s own delinquent behavior not only reflects prior risk factors but also further reinforces these factors which, in turn, cause future deviance (Matsueda, 1989; Raaijmakers et al., 2005). At the individual level, misconduct behavior might be a threat to one’s self-image and thus cause psychological discomfort if the delinquent behavior contradicts one’s internalized belief (Matsueda, 1989). One possible method to reduce that discomfort is to change one’s perception or belief to make them in line with that reflected by delinquent behavior, which might weaken one’s virtue in the long-run. To some extent, this proposition echoes the notion that “by experiencing or practicing virtue, individuals acquire virtue” (Narvaez and Lapsley, 2014, p. 230).

At the social interaction level, engaging in externalizing behavior would attenuate future bonds with parents, teachers, and peers, who constitute important aspects of social influence that fosters adolescents’ moral virtues (Narvaez, 2006; Narvaez and Lapsley, 2014). For instance, the children’s misconduct would result in harsher parenting that focuses more on controlling behavioral outcomes and interacting with the children in a unilateral way (Burke et al., 2008; Serbin et al., 2015). In turn, controlling parenting, poor parent-child communication and the lack of parental warmth are unfavorable conditions for the development of children’s more sociocentric orientation and moral character. For example, Lian et al. (2016) reported that negative parenting was a negative predictor of children’s moral virtues while positive parenting served as a positive predictor. The possible reason is that while the positive interaction between children and parents facilitates the children’s commitment to norms held by their parents and fosters the development of virtues expected by the parents, negative parenting leads to the children’s self-protective orientation that inhibits the development of virtues (Narvaez, 2006; Narvaez and Lapsley, 2014).

### Research Gaps

Based on the above literature review, four research gaps were identified. First, compared with the investigation on adolescent profile regarding moral virtues and the relationship between moral virtues and subjective well-being, there are very few studies adopting a virtue approach to examine how character virtues and adolescent externalizing behavior are associated with each other. In fact, promoting strengths of character has become one of the foci of the PYD programs (Catalano et al., 2002; Park and Peterson, 2006). In this sense, it is necessary to provide empirical evidence showing the positive impacts of moral virtues in reducing adolescents’ externalizing problems.

Second, the direction of the possible relationships between moral virtues and externalizing behavior has been under-researched. Although most of the extant literature assumes the causal effect of moral virtue on externalizing behavior, some studies directly or indirectly suggested a reversed causal effect (Matsueda, 1989; Raaijmakers et al., 2005), making it reasonable to expect the reciprocal causal effects between the two constructs. However, no empirical research has been done to test this possibility. This research gap is especially significant when taking into account the lack of large-scale longitudinal research in the related research field.

Third, while most research on adolescent problem behavior focused on actual behavior, such as addicted behavior or delinquency (de Kemp et al., 2009; Lian et al., 2016), very few studies have included delinquency and behavioral intention to engage in problem behavior in a single study. Adopting the indicators of actual behavior as well as future involvement helps to portray a more comprehensive picture of the relationships between moral competence and externalizing behavior.

Fourth, there is a lack of research based on non-Western populations, such as Chinese and African people. Given that Chinese people constitute nearly one-fifth of the world’s population, it can be argued that if the relationship between moral virtue and externalizing behavior is considered universal, it should be applicable to the Chinese adolescents (Shek, 2006). As such, there is a need to examine the relationship between moral virtues and externalizing behavior among Chinese adolescents.

### The Present Study

To fill the above research gaps, the present study investigated how moral competence defined as moral virtues is associated with externalizing behavior by employing a representative sample of Hong Kong adolescents over a 3-year period. Hong Kong was a British colony for nearly 150 years and has become a Special Administrative Region of the People’s Republic of China since 1997. Due to this unique history, Hong Kong has developed a relatively Westernized subculture that is different from the typical collectivist culture in Mainland China. Yet, 95% of Hong Kong’s population is Chinese who still firmly adhere to traditional Chinese culture and value core Chinese virtues (Yau and Smetana, 2003). Besides, empirical research found no difference between Hong Kong and Mainland adolescents regarding the development of Chinese virtues as well as the relationship between virtues and life satisfaction (Duan et al., 2012a). Therefore, we believe that findings obtained among Hong Kong Chinese adolescents are able to inform the relationship between moral virtues and externalizing behavior among Chinese adolescents.

There are two related research questions: (1) Are there any significant effects between moral competence and adolescent externalizing behavior? (2) If there are significant effects between the two constructs, what is the direction of the effects? Noteworthy, we have partially addressed the first question in elsewhere by examining the predicting effect of adolescent moral virtues on the *initial level* of and the *rate of change* in their externalizing behaviors across high school years (Shek and Zhu, 2018). Nevertheless, the reciprocal longitudinal effects between moral virtues and the occurrence of externalizing behaviors indexed by self-reported delinquency and intention to engage in delinquent behavior over time remained unknown.

**FIGURE 1:**
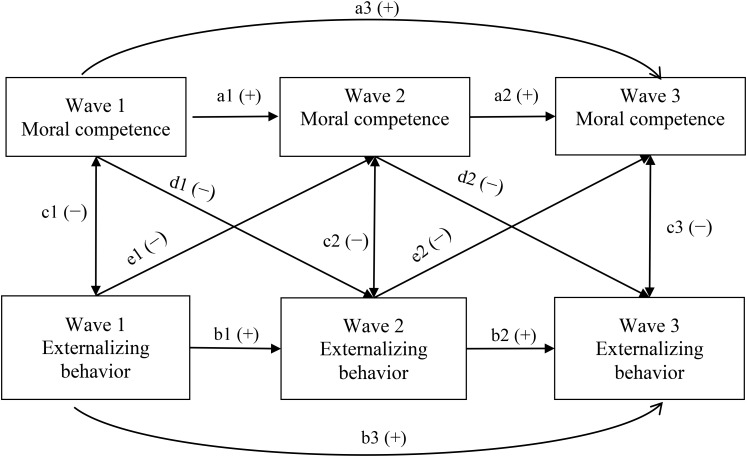
Autoregressive, cross-lagged panel path models. Model 1: No cross-lagged associations (d and e paths are both dropped). Model 2: Moral competence effects model (moral competence predicts externalizing behavior; e paths are dropped). Model 3: Externalizing behavior effects model (externalizing behavior predicts moral competence; d paths are dropped). Model 4: Reciprocal effects model (all paths are included).

Thus, to address the abovementioned two related questions comprehensively, the present study examined four competing cross-lagged panel path models to test four patterns of the relationships between the two constructs, respectively. Specifically, the first model (M1) is the no cross-lagged effects model where it is hypothesized that the two constructs do not impact each other directly, but share variance is caused by those unmeasured factors. The second model (M2) is the moral competence effects model which specifies the direct and negative effects of moral competence on externalizing behavior while the third one (M3) is the externalizing behavior effects model hypothesizing the direct and negative effects of externalizing behavior on moral competence. The last model (M4) is the reciprocal effects model in which the two constructs demonstrate reciprocal and negative impacts on each other. Figure 1 summaries these models. Based on the above literature review, we expected that the reciprocal effects model (M4) will show the best model fit.

As shown in Figure 1, in the cross-lagged panel models, both autoregressive effects that specify the stability of constructs across different waves, and the hypothesized cross-lagged effects of one construct on another from one time point to the next can be tested simultaneously. This feature helps to minimize the bias in estimating the hypothesized cross-lagged causal effects between different constructs.

## Materials and Methods

### Participants and Procedures

In a large-scale longitudinal project in Hong Kong that aimed at investigating Chinese adolescent developmental outcomes over time, 28 Chinese-speaking secondary schools were randomly selected to join the project. In 2009–2010 academic year, all Grade 7 (i.e., the 1st year of secondary school study) students in the participating schools were invited to complete a survey including multiple measures (e.g., delinquency, problem behavioral intention, moral competence, life satisfaction, etc.) and were followed up annually during their secondary school lives.

The longitudinal project was approved by the Human Subjects Ethics Sub-committee (HSESC) of The Hong Kong Polytechnic University. Before the Wave 1 data collection, informed written consent was obtained from the participating schools and the parents of the student participants. They were fully explained that the information provided by the students would be used only for academic research purposes and be kept confidential, and the students could withdraw from the project whenever they want. At the beginning of each wave of data collection, informed written consent was also obtained from all participating students after they were well informed about the principles of voluntarism and confidentiality. In each wave of data collection, using a paper-and-pencil questionnaire containing validated instruments, the trained research staff administrated the survey in the quiet classrooms in each participating school.

The research team used different data obtained from the longitudinal project to address different research questions (Shek and Liu, 2014; Shek and Lin, 2016b, 2017; Yu and Shek, 2017; Shek and Zhu, 2018), including the aforementioned one which examined the relationship between two positive traits (i.e., “moral competence” and “spirituality”) and the level of as well as the rate of change in adolescent externalizing behavior (Shek and Zhu, 2018). Noteworthy, based on 3-wave data covering the participants’ junior secondary years, the present study aimed to address the possibility of reciprocal causal relationships between moral competence and externalizing behavior, which is basically different from the foci of our previous work, which only focused on the impact of moral competence on the initial level and growth of delinquency.

Specifically, Wave 1 data were collected when the participants just started their secondary school study while Wave 2 and Wave 3 data were collected when the students entered the 2nd and the 3rd year of their secondary school lives, respectively. At Wave 1, the sample included 3,328 Grade 7 students (Age = 12.59 ± 0.74 years), among whom 52.1% (*n* = 1,735) were boys, 47.6% (*n* = 1,584) were girls and 0.3% (*n* = 9) did not provide information on their gender. As shown in Table 1, a total of 2,905, and 2,860 participants remained in the project at Wave 2 and 3, respectively. The attribution rate at Wave 2 (12.7%) and Wave 3 (14.1%) were acceptable. The full sample including the 3,328 participants was utilized in the present study.

**Table 1:** Data profile across the three waves.

	Wave 1	%	Wave 2	%	Wave 3	%
*N* (Participants)	3,328		2,905		2,860	
Grade level	Grade 7		Grade 8		Grade 9	
Gender						
Male (*n*)	1,735	52.1	1,461	50.3	1,438	50.3
Male (age)	12.63 ± 0.78		13.63 ± 0.72		14.59 ± 0.70	
Female	1,584	47.6	1,440	49.6	1,417	49.5
Female (age)	12.55 ± 0.70		13.54 ± 0.68		14.50 ± 0.66	
Age range	10–18		11–17		13–18	
Age (*M* ±*SD*)	12.59 ± 0.74		13.59 ± 0.70		14.55 ± 0.68	
Attrition rate (%)			12.70		14.10	


*Grade 7 is the 1st year of secondary school study; Grade 8 is the 2nd year of secondary school study; Grade 9 is the 3rd year of secondary school study.*

### Instruments

The questionnaire used in the project included multiple measures (Shek and Ma, 2014). Moral competence and the two measures of externalizing behavior (i.e., delinquency and problem behavioral intention) were the foci of the present study.

#### Moral Competence

Moral competence in the present study was assessed by one subscale of a shortened version of scale entitled “Chinese Positive Youth Development Scale” (CPYDS), which was specifically developed for Chinese adolescents. The scale included 15 subscales, all of which possessed acceptable internal consistency (Cronbach’s αs ranged between 0.63 to 0.91), were able to discriminate adolescents with well adjustment form those who had poor adjustment, and were positively associated with other measures of well-being (e.g., academic achievement and life satisfaction) while negatively related to problematic behaviors (e.g., substance abuse) (Shek et al., 2007; Shek and Ma, 2010). The CPYDS has been widely employed to assess positive developmental characteristics among Chinese youths (Shek and Lin, 2016a; Shek et al., 2017). To minimize participant fatigue in completing a long questionnaire, a shortened version of the CPYDS was adopted in the longitudinal project by selecting three items possessing the highest factor loadings in each of the 15 original subscales, which maximize the validity and reliability of the shortened version (Shek and Ma, 2010). The three items used to measure moral competence were: (1) “I have a high moral expectation about my behavior”; (2) “I will fulfill my promise”; and (3) “I have the habit of self-evaluation.” Conceptually, these three items assessed the aforementioned critical elements of Chinese virtues in Confucianism and echoed the character strengths in the VIA model (Park and Peterson, 2006). The participants indicated the degree of their agreement with the three statements on a 6-point rating scale (1 = “strongly disagree,” 6 = “strongly agree”). An average score across the three items was computed to represent moral competence.

#### Delinquency

Delinquency was assessed by asking the participants to indicate the frequency of doing different delinquent acts in the past 12 months. The delinquent acts included “stealing,” “cheating others,” “truancy,” “running away from home,” “damaging others’ properties,” “beating others,” “gang fighting,” “speaking foul language,” “staying away from home overnight without parental consent,” “bullying,” and “trespassing.” A 7-point scale was used and a higher score indicated a higher level of delinquency. An average score across all items was calculated.

#### Problem Behavioral Intention

This construct was assessed using a 5-item scale. On a 4-point scale (1 = “absolutely will not,” 4 = “absolutely will”), the participants indicated their willingness to engage in five forms of problem behavior (i.e., “smoking,” “drinking,” “gambling,” “drug abuse,” and “having sexual intercourse”) in the next 2 years. An average was also calculated.

#### Validity and Reliability of Measures

Confirmatory Factor Analyses (CFA) were used to test measurement models across the three assessment occasions (Waves 1–3). At each wave, the model included the three key constructs (i.e., moral competence, delinquency, and problem behavioral intention) indexed by respective items. In these measurement models, all related measures were considered unidimensional according to the hypothesized structure of the measures. Results of CFA revealed that the three measurement models fit the data reasonably well (i.e., GFI > 0.90; RMSEA < 0.08; SRMR < 0.08), although one index fell below the ideal value (i.e., CFI ranged between 0.85 and 0.88) (Hair et al., 2006; Shek and Yu, 2014). Based on factor loadings derived from CFA, we calculated Average Variance Extracted (AVE) and Composite Reliability (CR) for each construct to indicate convergence and reliability (Fornell and Larcker, 1981; Hair et al., 2006). As shown in Table 2, CRs ranged from 0.73 to 0.83, which exceeded 0.70 rule of thumb, suggesting adequate reliability (Fornell and Larcker, 1981). According to Fornell and Larcker (1981), 0.50 is considered the threshold for AVE, meaning that on average, more variance (over 50%) of the observable factors is explained by the latent construct imposed than residuals. As AVE represents the average squared factor loading, an AVE of 0.50 or higher generally indicates that the average factor loading reaches at least 0.71 (0.71^2^ = 0.5041). Nevertheless, according to some researchers (e.g., Hair et al., 2006; Li et al., 2018), an acceptable factor loading should be at least 0.30 to 0.50. In this sense, an AVE of or above 0.25 (0.50^2^ = 0.25) could still be seen as acceptable. Based on this argument, the AVEs of the present constructs were equal to or higher than 0.25 (see Table 2). Besides, the average factor loadings were of or above 0.50 as well (see Table 2). Finally, the Cronbach’s α and mean inter-item correlation of each measure suggested acceptable internal consistency (see Table 2). Taken together, these results suggested that the three measures possessed acceptable factorial validity and reliability in the present study.

**Table 2:** Validity and reliability of scales across the three waves.

Scale	Wave	Average factor loading	Average variance extracted	Composite reliability	Cronbach’s α	Mean inter-item correlation
Moral competence	Wave 1	0.69	0.48	0.73	0.74	0.48
	Wave 2	0.70	0.50	0.75	0.75	0.50
	Wave 3	0.70	0.49	0.74	0.74	0.49
Delinquency	Wave 1	0.52	0.28	0.80	0.81	0.27
	Wave 2	0.56	0.32	0.83	0.84	0.33
	Wave 3	0.50	0.25	0.78	0.79	0.25
Problem behavioral intention	Wave 1	0.61	0.38	0.75	0.74	0.36
	Wave 2	0.64	0.41	0.77	0.76	0.38
	Wave 3	0.60	0.36	0.74	0.72	0.34


### Attrition Analyses

We compared the participants of a matched group with complete data across the three waves (*N* = 2,669) with those who dropped out after Wave 1. Compared with the dropouts, those who in the matched group were slightly younger, included a smaller proportion of boys and had a higher level of moral competence and a lower level of externalizing behavior at Wave 1. To deal with the systematic attrition, we imputed the missing values of moral competence and the two indicators of externalizing behavior using the multiple imputation strategy. We applied the “Predictive Mean Matching” option in using the “multiple imputation” module of SPSS and performed forty times of imputation (i.e., forty imputed data sets were obtained) (Asendorpf et al., 2014).

Further statistical analyses were conducted employing the 40 imputed data sets and the original one separately. A pooled result was calculated for each statistical parameter by averaging the corresponding values across the 40 imputation samples (Rubin, 2004). Comparisons between the pooled results and the results obtained from the original data set yielded similar findings, suggesting that the attrition did not cause significant bias in the present study. Therefore, the pooled results were reported in the results section below for correlations and the cross-lagged path analyses. The above procedure used to investigate and minimize the bias caused by attrition is highly recommended for the longitudinal research and has been widely used recently (e.g., Asendorpf et al., 2014; Huijbers et al., 2015).

### Data Analysis

We first conducted descriptive statistics and correlation analyses using SPSS 23.0 (IBM Corp., Somers, NY, United States). With AMOS 23.0 (IBM Corp., Somers, NY, United States), the cross-lagged path analyses were then performed based on the total sample to compare the four models as depicted in Figure 1. The chi-square differences tests were utilized to compare the fit of the competing models. According to Byrne (2016), change in chi-square value can be regarded as a valid indicator of relative model fit.

## Results

The descriptive statistics of all the related variables were summarized in Tables 1, 2. Table 3 demonstrates the cross-sectional and longitudinal correlations among the considered variables. As expected, moral competence was negatively correlated with externalizing behavior indicators, both concurrently and longitudinally. The results also showed that girls tended to have a higher level of moral competence and a lower level of externalizing problems than boys.

**Table 3:** Correlations among variables.

Variables		Range	*M*	*SD*	1	2	3	4	5	6	7	8	9
(1)	W1 Moral competence	1–6	4.37	0.91	–								
(2)	W2 Moral competence	1–6	4.41	0.85	0.49^∗∗∗^	–							
(3)	W3 Moral competence	1–6	4.46	0.81	0.42^∗∗∗^	0.53^∗∗∗^	–						
(4)	W1 Delinquency	0–6	0.43	0.51	–0.32^∗∗∗^	–0.23^∗∗∗^	–0.19^∗∗∗^	–					
(5)	W2 Delinquency	0–6	0.50	0.59	–0.27^∗∗∗^	–0.30^∗∗∗^	–0.22^∗∗∗^	0.58^∗∗∗^	–				
(6)	W3 Delinquency	0–6	0.48	0.53	–0.22^∗∗∗^	–0.24^∗∗∗^	–0.23^∗∗∗^	0.48^∗∗∗^	0.60^∗∗∗^	–			
(7)	W1 Problem behavioral intention	1–4	1.26	0.39	–0.21^∗∗∗^	–0.16^∗∗∗^	–0.11^∗∗∗^	0.49^∗∗∗^	0.38^∗∗∗^	0.28^∗∗∗^			
(8)	W2 Problem behavioral intention	1–4	1.34	0.45	–0.18^∗∗∗^	–0.23^∗∗∗^	–0.15^∗∗∗^	0.38^∗∗∗^	0.52^∗∗∗^	0.36^∗∗∗^	0.52^∗∗∗^		
(9)	W3 Problem behavioral intention	1–4	1.37	0.46	–0.20^∗∗∗^	–0.21^∗∗∗^	–0.20^∗∗∗^	0.35^∗∗∗^	0.39^∗∗∗^	0.51^∗∗∗^	0.41^∗∗∗^	0.55^∗∗∗^	
(10)	Gender^a^				–0.12^∗∗∗^	–0.11^∗∗∗^	–0.09^∗∗∗^	0.07^∗∗∗^	0.08^∗∗∗^	0.11^∗∗∗^	0.07^∗∗∗^	0.05^∗∗^	0.08^∗∗∗^


*^a^Female = 1; Male = 2. W1, Wave 1; W2, Wave 2; W3, Wave 3. ^∗∗^p < 0.01, ^∗∗∗^p < 0.001.*

Table 4 presents the results of cross-lagged path analyses. First, the no cross-lagged effects model (M1) without cross-lagged associations between moral competence and delinquency was compared to the moral competence effects model (M2) with the cross-lagged effects of moral competence on delinquency. The results showed that M2 had better model fit [Δχ^2^_(2)_ = 24.40, *p* < 0.001]. Second, the delinquency effects model (M3) with delinquency at an earlier time point impacting on moral competence at the later time point fit the data better than M2 [Δχ^2^_(2)_ = 31.86, *p* < 0.001]. Third, the reciprocal effects model (M4) yielded a better model fit than M1 [Δχ^2^_(4)_ = 50.57, *p* < 0.001], M2 [Δχ^2^_(2)_ = 26.17, *p* < 0.001], and M3 [Δχ^2^_(2)_ = 18.71, *p* < 0.001]. Therefore, the reciprocal effects model (M4) was supported by the data, suggesting that reciprocal causal relationships between moral competence and delinquency were present.

**Table 4:** Model fit indexes and comparison for different models of the relationship between moral competence and delinquency.

Models	χ^2^	*df*	CFI	IFI	TLI	RMSEA (90% CI)	Model comparison	Δχ^2^	Δ*df*	*p*
M1: No cross-lagged effects	53.09	6	0.93	0.93	0.82	0.063 (0.048, 0.078)				
M2: Moral competence effects	28.69	4	0.96	0.96	0.86	0.055 (0.037, 0.075)	M1 vs. M2	24.40	2	<0.001
M3: Delinquency effects	21.23	4	0.97	0.97	0.90	0.046 (0.028, 0.066)	M1 vs. M3	31.86	2	<0.001
M4: Reciprocal effects	2.52	2	0.99	0.99	0.99	0.011 (0.000, 0.047)	M1 vs. M4	50.57	4	<0.001
							M2 vs. M4	26.17	2	<0.001
							M3 vs. M4	18.71	2	<0.001


Similarly, as demonstrated in Table 5, M4 specifying the reciprocal causal relationships between moral competence and problem behavioral intention was also best supported by the data compared to other three competing models. Therefore, we concluded that there are reciprocal causal relationships between moral competence and externalizing behavior.

**Table 5:** Model fit indexes and comparison for different models of the relationship between moral competence and problem behavioral intention.

Models	χ^2^	*df*	CFI	IFI	TLI	RMSEA (90% CI)	Model comparison	Δχ^2^	Δ*df*	*p*
M1: No cross-lagged effects	77.95	6	0.97	0.97	0.91	0.077 (0.062, 0.093)				
M2: Moral competence effects	27.28	4	0.99	0.99	0.95	0.054 (0.036, 0.074)	M1 vs. M2	50.67	2	<0.001
M3: Problem behavioral intention effects	62.39	4	0.98	0.98	0.89	0.085 (0.067, 0.104)	M1 vs. M3	15.56	2	<0.001
M4: Reciprocal effects	12.74	2	0.99	0.99	0.96	0.052 (0.027, 0.080)	M1 vs. M4	65.21	4	<0.001
							M2 vs. M4	14.54	2	<0.001
							M3 vs. M4	49.65	2	<0.001


**FIGURE 2:**
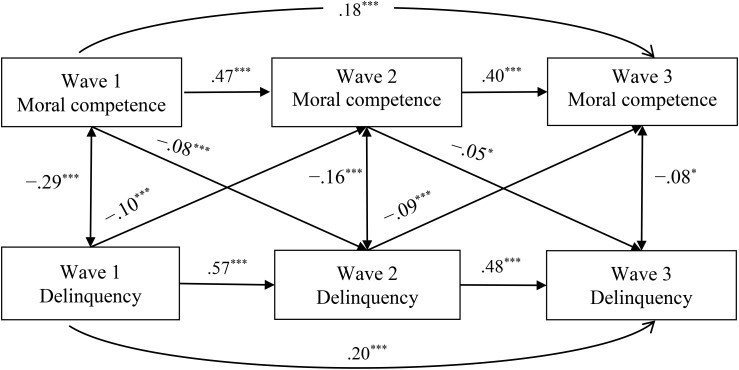
Standardized path coefficients for the reciprocal associations model between moral competence and delinquency. χ^2^_(2)_ = 2.52, *p* = 0.28, CFI = 0.99, IFI = 0.99, TLI = 0.99, RMSEA = 0.011. ^∗^*p* < 0.05, ^∗∗^*p* < 0.01, ^∗∗∗^*p* < 0.001.

Figure 2, 3 further demonstrate the path coefficients of the supported reciprocal effects model concerning delinquency [χ^2^_(2)_ = 2.52, CFI = 0.99, IFI = 0.99, TLI = 0.99, RMSEA = 0.011] and problem behavioral intention [χ^2^_(2)_ = 12.74, CFI = 0.99, IFI = 0.99, TLI = 0.96, RMSEA = 0.052], respectively.

**FIGURE 3:**
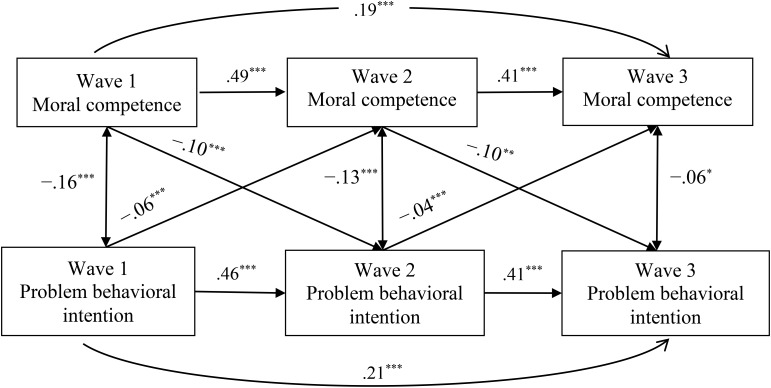
Standardized path coefficients for the reciprocal associations model between moral competence and problem behavioral intention. χ^2^_(2)_ = 12.74, *p* = 0.002, CFI = 0.99, IFI = 0.99, TLI = 0.96, RMSEA = 0.052. ^∗^*p* < 0.05, ^∗∗^*p* < 0.01, ^∗∗∗^*p* < 0.001.

As showed in Figure 2, adolescent moral competence at Wave 1 and Wave 2 had a negative longitudinal effect on their delinquency at Wave 2 (β = -0.08, *p* < 0.001) and Wave 3 (β = -0.05, *p* = 0.02), respectively. Similarly, the participants’ delinquency at Wave 1 and Wave 2 negatively predicted their moral competence at Wave 2 (β = -0.10, *p* < 0.001) and Wave 3 (β = -0.09, *p* < 0.001), respectively. These results indicated that a lower level of moral competence in adolescents would cause a higher level of future delinquency and vice versa.

Likewise, adolescents’ moral competence at Wave 1 and Wave 2 negatively predicted their intention to engage in problem behavior at Wave 2 (β = -0.10, *p* < 0.001) and Wave 3 (β = -0.10, *p* = 0.02), respectively (see Figure 3). Meanwhile, the participants’ problem behavioral intention at Wave 1 and Wave 2 also negatively predicted their moral competence at Wave 2 (β = -0.06, *p* < 0.001) and Wave 3 (β = -0.04, *p* < 0.001), respectively. These results suggested that a lower level of moral competence in adolescents would cause a higher level of future intention to engage in problem behavior and vice versa.

## Discussion

Based on the three-wave cross-lagged panel design, the present study showed the reciprocal causal effects between moral competence defined by virtues and adolescent externalizing behavior during junior secondary school years. Specifically, the level of moral competence at Wave 1 negatively predicted externalizing behavior at Wave 2 after the baseline status was controlled. Similarly, the level of adolescent externalizing behavior at Wave 1 inversely affected moral competence at Wave 2. The same reciprocal relationships between the two constructs were observed from Wave 2 to Wave 3. These results indicate that moral competence influences externalizing behavior as well as being impacted by it.

The negative longitudinal impact of moral virtues on delinquency found in the present study is consistent with the negative association between other conceptions of morality such as moral judgment and delinquency identified in previous cross-sectional studies (Stams et al., 2006; Cheng, 2014). These findings suggest that to some extent, different elements of morality may exert a similar influence on adolescent delinquent behavior (Park and Peterson, 2006; Kulju et al., 2016). Furthermore, rather than a “snapshot” of the inverse association between moral competence and delinquency at one single time point, the present study further displayed its stability over time. Adolescence is marked by rapid development in cognitive, emotional and social abilities, which help them develop their self-concept and a mature self-identity. During this process, adolescent moral character constitutes one important dimension that will be integrated with other components of self-identity (Levine et al., 1992; Hardy et al., 2014). Generally speaking, moral character embedded in adolescents’ identity system connote the development of socially valued motivations, beliefs, attitudes, and norms; all could drive the adolescents to do socially desirable behavior as doing so is an important part of whom they are.

Theoretical assertions and empirical findings support the conclusion that moral virtues are essential for an individual to thrive. Specifically, good characters and virtues have been found to be associated with well-being and better psychosocial adjustment across age groups ranging from young children to adults (Seligman, 2002; Park and Peterson, 2006; Hausler et al., 2017; Baumann et al., 2019; Shoshani, 2019). This conclusion is also supported in the Chinese context, mainly among university students and adults (Duan and Ho, 2018; Zhang and Chen, 2018). In view of the lack of empirical evidence among Chinese adolescents, particularly regarding the relationship between moral virtues and problem behaviors, the present finding, which showed the negative longitudinal influence of moral virtues on externalizing behavior, provided further support for the argument in positive psychology that promoting one’s character strengths is a solid way to construct one’s good life characterized by well-being and staying away from delinquent behavior.

The present study also revealed a negative longitudinal effect of externalizing behavior on moral virtues across the early adolescent years. This finding supports our hypothesis that the development of an adolescent’s virtues would be affected by their behavior (or even the intention to engage in certain behavior) as well. On one hand, adolescents with more externalizing behaviors are less likely to practice character strength or virtue than their peers who had less such behaviors. Practicing positive character attributes has been proved to be beneficial for one’s healthy development in terms of positive and clear self-identity, higher self-esteem, better performance, higher life satisfaction, and better relations (Huber et al., 2017; Littman-Ovadia et al., 2017; Zhang and Chen, 2018). As a result, we may infer that externalizing behaviors, in fact, hinder adolescents’ practice of character strength, which in turn hinder further development of virtues. Further research could test this possibility by examining the relationships among externalizing behaviors, use of character strengths, and the further development of moral virtues.

On the other hand, because adolescents’ identity and value system are not mature enough and thus are subject to change, their beliefs and reasoning ability which can be regarded as certain aspects of identity were found to be susceptible to “external” pressures including one’s own behavior (Matsueda, 1989; Raaijmakers et al., 2005). This mechanism could also be applicable to the development of adolescents’ virtues which kindly constitute another dimension of the identity system. Hence, the higher level of involvement in delinquent activities presents more pressure to one’s self-perception and identity, leading to certain changes of thoughts, attitudes, and beliefs toward egocentric orientation (Gini et al., 2014), which in the long-run would hinder the development of prosocial orientation and strong characters. In this line of reasoning, the actual behavior may cause more pressure to one’s development of moral competence than does the behavioral intention which may not lead to actual behavior. This may be the reason that in the present study, the previous delinquent behavior showed stronger effects on future moral competence than did problem behavioral intention. As no previous research has examined the causal effect of externalizing behavior on moral virtues, the novel findings obviously demand replications in other parts of the world.

The panel design used in the present study is a rigorous method to establish the causality and reciprocity of the relationships between moral virtues and externalizing behavior. However, the magnitudes of the reciprocal effects observed in the present study appeared small, albeit statistically significant, stable and systematic. The large sample may also easily generate significant findings with small effect sizes. However, as stressed by Raaijmakers et al. (2005, p. 256), it is not uncommon that the longitudinal predicting effects on delinquency are “less than half the size of corresponding coefficients in cross-sectional models.” In fact, Raaijmakers et al. (2005) also reported small effects regarding the reciprocal relationship between moral reasoning and delinquency. Furthermore, it is very difficult, if not impossible, to establish large effect sizes with respect to the prediction of psychosocial constructs on future behavior, because the inclusion of previous behavior as a predictor would substantially account for the variance of future behavior, leaving little room to show the influence of other factors (Raaijmakers et al., 2005). Given that the present study is a pioneer attempt to investigate the causal relationships between moral virtues and externalizing behavior, future replications and verifications of the findings in other populations and developmental stages are warranted.

To sum up, the present study has several advances. First, moral competence was assessed by virtues, which is an important but under-researched component in the field on the relationship between morality and adolescent behavior (Catalano et al., 2002; Park and Peterson, 2006). Second, externalizing behavior was indexed by not only actual delinquent behavior but also adolescents’ intention to engage in problem behavior in the future. Third, the study used longitudinal data which can effectively establish the direction of effects between moral competence and externalizing behavior. Fourth, the reciprocal relationships between moral competence and the two measures of externalizing behavior were identified, which deepen our understanding of the causes and consequences of the (lack of) development of moral virtues and externalizing behavior in terms of actual delinquency as well as the intention to engage in delinquency. Fifth, we employed a large representative sample of Chinese adolescents, which helps to shed light on the universality of the relationships between the two constructs under investigation.

Nevertheless, some limitations of the present study should be noted. The first limitation concerns the self-report measures for moral competence and the two indicators of externalizing behavior. Self-report measures might lead to socially desirable responses (e.g., lower level of externalizing behavior and higher level of moral competence), which might in turn result in artificially large negative associations between externalizing behavior and moral competence. It is methodologically preferable if future studies could involve different informants, such as peers, parents, and teachers. However, it can be counter-argued that adolescents may know their own situations and experiences better than others and self-report measures are in fact widely adopted in research on adolescents (Thornberry and Krohn, 2003). Besides, the operational definition of “problem behavior” in the measure of “problem behavior intention” could be further sharpened. While problem behavior (i.e., smoking, drinking, gambling, drug abuse, and having sexual intercourse in high school years) covered in this study may have different interpretations across cultures, they are commonly regarded as high-risk adolescent behavior. For example, while we regard sexual maturity in adolescent years as a normative development, we included “having sexual intercourse” on the list of problem behavior because in Chinese society, parents and teachers commonly hold a negative view toward sexual intercourse in the high school years. Another related issue is the usage of the paper-and-pencil questionnaire administrated in classrooms. Although collecting data using computer-based survey has merit such as higher cost-effectiveness and greater security, it has higher requirements for computer facilities in the participating schools and there are practical difficulties in arranging students to complete the questionnaire in the computer room. Besides, paper-and-pencil administration can reduce attribution rate and missing values, which is especially essential in a longitudinal study (Wyrick and Bond, 2011). As a result, the paper-and-pencil survey could be considered a good choice in the present school-based longitudinal study.

It will also be helpful to minimize social desirability if using an objective measure of delinquency, such as the known-group comparison (e.g., officially identified delinquents versus non-delinquent age-mates) adopted in the studies on moral reasoning and delinquency (see Stams et al., 2006). Unfortunately, the known-group comparison is difficult, if not entirely impossible, to be utilized in a longitudinal study. This may constitute one of the reasons that almost all studies adopting official records of delinquency were cross-sectional (Stams et al., 2006). This point has also been mentioned in previous work (Shek and Zhu, 2018). It is also worth noting that some strategies were used in the present study to reduce the bias caused by social desirability. For instance, the participants were clearly instructed not to communicate with classmates and were well informed with the principle of anonymity before data collection at each time point. In fact, the self-report measures have been commonly used to examine the relationships between adolescent delinquency and morality in the existing literature, especially in longitudinal studies (Raaijmakers et al., 2005; Emler and Tarry, 2007).

The second limitation is related to the conceptualization of moral competence. Although we had good reasons to consider virtues as an important component of morality that has been traditionally under-researched especially in the Chinese context, we need to keep in mind that morality consists of multiple facets such as virtues, moral reasoning ability, moral beliefs and moral self-concept (Cheng, 2014; Kulju et al., 2016). While it is important to understand how moral virtues itself is linked to adolescent behavior, it is worth to deepen our understanding by looking at the interactions between different constructs related to morality.

The third limitation pertains to the investigation period. The present study covered a 3-year period of the junior secondary school stage which represents early adolescence. Given that limited literature is available regarding the reciprocal relationship between moral virtues and externalizing behavior, future studies need to replicate the present findings by covering an even longer period. By doing so, stability or changes of the relational pattern across the life span could be uncovered.

Fourth, the present findings were obtained from Hong Kong Chinese adolescents. Despite the large sample size, they may not represent all Chinese, due to potential different subcultures in Hong Kong and Mainland China. For example, young people in Hong Kong may be more oriented toward Western values such as individualism while Mainland Chinese youths are more prone to traditional Confucian values such as collectivism. Empirical research found that Hong Kong Chinese adolescents expressed conflicts with parents differently from their Mainland Chinese counterparts (Yau and Smetana, 2003). Nevertheless, adolescents in the two Chinese communities did not differ from each other regarding the development of Chinese virtues and the relationship between virtues and well-being (Duan et al., 2012a). The possible reason is that the core Chinese virtues are valued in the two subcultures. In this respect, it is reasonable to expect a similar relational pattern between moral virtues and externalizing behavior in Hong Kong and Mainland China. Concerning the severe lack of related research among Chinese adolescents, the present findings add value to the literature. Yet, it is necessary to conduct more studies in other Chinese communities to further verify the universality of the present findings.

Despite the above limitations, the present study addresses the research gaps outlined earlier. Our pioneer findings reveal the existence of dynamic reciprocal relationships between moral competence and externalizing behavior during early adolescence in a non-Western context which has important theoretical significance. There are mainly two important educational implications of the present study. First, the present findings imply that promoting virtues may help reduce externalizing behavior such as delinquency. On one hand, this further consolidates the foundation of preventing adolescent problem behavior through promoting positive youth development, which includes moral competence as one of the 15 important psychosocial components (Catalano et al., 2002; Park and Peterson, 2006). In addition, researchers and educators can specifically develop and implement moral education programs that incorporate core Chinese virtues to help adolescents recognize, develop, and apply their moral virtues. Second, the present finding echoes previous theoretical suggestion that practicing virtue by doing good things could be a way to promote the development of virtues in the long run (Narvaez and Lapsley, 2014). Therefore, parents and teachers should encourage students to stay far away from delinquent behaviors but actively participate in meaningful activities such as providing volunteer services. Additionally, families, schools, communities, and even the society should create a supportive environment to guide adolescents to do good things.

## Author Contributions

DS designed the project and contributed to all steps of the work. XZ contributed to the idea construction and data interpretation of the work, drafted the work and revised it based on the critical comments provided by DS. DS and XZ approved the final version of the manuscript and agree to be accountable for all aspects of the work in ensuring that questions related to the accuracy or integrity of any part of the work are appropriately investigated and resolved.

## Conflict of Interest Statement

The authors declare that the research was conducted in the absence of any commercial or financial relationships that could be construed as a potential conflict of interest.
